# Role of autotaxin in systemic lupus erythematosus

**DOI:** 10.3389/fmed.2023.1166343

**Published:** 2023-04-04

**Authors:** Yumi Tsuchida, Hirofumi Shoda, Tetsuji Sawada, Keishi Fujio

**Affiliations:** ^1^Department of Allergy and Rheumatology, Graduate School of Medicine, The University of Tokyo, Tokyo, Japan; ^2^Department of Rheumatology, Tokyo Medical University Hospital, Tokyo, Japan

**Keywords:** autotaxin (ATX), SLE, lysophosphatidic acid, lysophospholipids, type I interferons

## Abstract

Systemic lupus erythematosus (SLE) is a prototypic systemic autoimmune disease characterized by the production of various autoantibodies and deposition of immune complexes. SLE is a heterogenous disease, and the pattern of organ involvement and response to treatment differs significantly among patients. Novel biological markers are necessary to assess the extent of organ involvement and predict treatment response in SLE. Lysophosphatidic acid is a lysophospholipid involved in various biological processes, and autotaxin (ATX), which catalyzes the production of lysophosphatidic acid in the extracellular space, has gained attention in various diseases as a potential biomarker. The concentration of ATX is increased in the serum and urine of patients with SLE and lupus nephritis. Recent evidence suggests that ATX produced by plasmacytoid dendritic cells may play an important role in the immune system and pathogenesis of SLE. Furthermore, the production of ATX is associated with type I interferons, a key cytokine in SLE pathogenesis, and ATX may be a potential biomarker and key molecule in SLE.

## 1. Introduction

Systemic lupus erythematosus (SLE) is a systemic autoimmune disease, common in young women. SLE is characterized by the production of various autoantibodies, such as anti-nuclear and anti-dsDNA antibodies, and deposition of immune complexes. SLE can affect various organs including the skin, heart, kidneys, and central nervous system. Current treatment for SLE includes antimalarials, corticosteroids, immunosuppressants, and biologics; however, it is far from perfect, and many patients do not achieve an adequate response or experience side effects.

SLE is a heterogenous disease and the pattern of organ involvement and response to treatment differs significantly among patients. Various biological markers, such as complement levels, anti-dsDNA antibody titers, and urine tests, are used in clinical settings to assess disease activity of SLE (1). Different autoantibodies have been reported to be associated with certain clinical features of SLE, for example, the association of anti-ribosomal P antibodies with neuropsychiatric lupus and lupus hepatitis (2). Other novel markers are also being investigated (3), for example, monocyte chemoattractant protein-1 levels in urine have been reported to correlate with the activity and prognosis of lupus nephritis (4–6). Serum interferon levels have been associated with disease activity (7, 8). However, these biomarkers are not sufficient to assess the extent of disease and predict treatment response, and it is pertinent to find markers that can stratify patients to provide treatment most effective for each individual.

Recently, lysophosphatidic acid (LPA) and autotaxin (ATX), an enzyme that catalyzes the production of LPA, have recently gained attention in many fields. Many ATX inhibitors are being developed as potential therapeutic agents for cancer and idiopathic pulmonary fibrosis, and there are currently several clinical trials underway (9–11). Recently, a role for the ATX-LPA axis in SLE has also been reported. In this review, we will discuss the roles of LPA and ATX and whether they are their possible biological markers in SLE.

## 2. Lysophospholipids and the autotaxin–lysophosphatidic acid axis

Lysophospholipids are lipids with one carbon chain and a polar head group. They are classified into two groups, lysoglycerophospholipids and lysosphingolipids. *Via* signaling through G protein-coupled receptors, lysophospholipids play an important role in regulating cell function. LPA and sphingosine-1-phosphate are among the most well-studied lysophospholipids involved in cell signaling.

**Figure 1:**
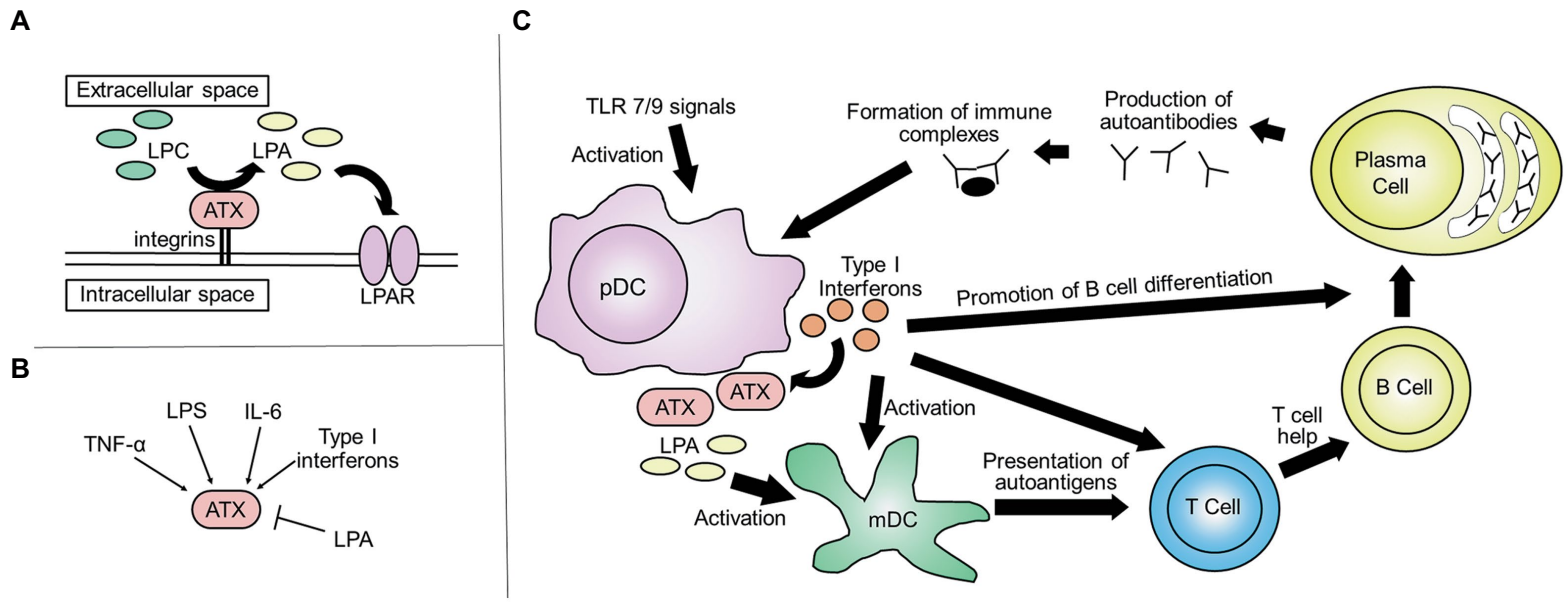
An Overview of ATX. **(A)** The ATX-LPA axis: ATX is a lysophospholipase that mainly catalyzes the production of LPA from lysoglycerophospholipids, such as lysophosphatidylcholine. (LPC, lysophosphatidylcholine; ATX, autotaxin; LPA, lysophosphatidic acid; LPAR, LPA receptor). **(B)** Regulation of ATX expression: the expression of ATX is induced by TNF, IL-6, LPS, and type I interferons and inhibited by LPA. (TNF, tumor necrosis factor; IL-6, interleukin-6; LPS, lipopolysaccharide). **(C)** ATX and the pathogenesis of SLE: pDCs are activated by TLR7 and TLR9 signals, resulting in the production of type I interferons. Type I interferons induce the production of ATX and thus the production of LPA. This results in the activation of mDCs, which present autoantigens to T cells and activate them. Together, this leads to the maturation of B cells and the production of autoantibodies. Immune complexes further enhance the activation of pDCs, resulting in a positive feedback loop, contributing to the organ damage in SLE (pDC, plasmacytoid dendritic cell; TLR, toll-like receptors; mDC, myeloid dendritic cell).

LPA transduces signals through LPA receptors (LPAR1–6) and is involved in various biological processes, including cell migration, proliferation, and aggregation of platelets (12). Under physiological conditions, ATX is the lysophospholipase mainly responsible for the production of LPA in blood and catalyzes the production of LPA from lysoglycerophospholipids, such as lysophosphatidylcholine (12) (Figure 1A). Five ATX isoforms have been reported, all of which exhibit lysophospholipase D activity. ATXβ and ATXγ are the isoforms mainly expressed in peripheral tissue and the central nervous system, respectively (13). ATX is abundantly expressed in adipose tissue. In mice, adipose tissue-specific knockout of ATX significantly decreased the concentration of LPA in plasma, suggesting that adipose tissue is an important source of ATX in blood (14). ATX is also highly expressed in the central nervous and reproductive systems, as well as in lymphoid tissues (13, 15). In the extracellular space, ATX binds to integrins, preventing its clearance and allowing for localized LPA production (16, 17). Tumor necrosis factor (TNF), interleukin-6 (IL-6), and lipopolysaccharide, as well as type I interferons, have been reported to induce the expression of ATX, while the expression of ATX is inhibited by LPA in a negative feedback loop (15, 18) (Figure 1B).

## 3. Measuring lysophosphatidic acid and autotaxin concentrations

Many studies have investigated the concentrations of LPA and ATX in clinical specimens. Both LPA and ATX are abundant in the blood. Concentrations of LPA in the plasma of healthy individuals vary significantly among reports, ranging from 40–60 nM (19) to 120 nM (20). Concentrations as high as 0.1–6.3 μM have also been reported (21). Significant differences among these reports are thought to reflect the difficulty of accurately measuring LPA concentrations. That is, the concentration of LPA increases rapidly after blood collection due to its production *in vitro*. Therefore, to accurately measure the concentration of LPA in clinical specimens, samples must be handled carefully to avoid the production and degradation of LPA (19, 20). Further, its concentration is slightly higher in females (22).

The concentration of ATX in serum has been reported to be closely correlated with that of LPA and is more stable (22–24); thus, the measurement of ATX may serve as a more practical marker. The serum concentration of ATX is also higher in females than in males: 0.625–1.323 mg/L and 0.438–0.914 mg/L, respectively (9). The serum level of ATX increases during pregnancy (25) and various diseases, which will be discussed in section 4. Serum concentration of ATX may be affected by treatment with steroids (26, 27).

## 4. Autotaxin and human diseases

Reflecting the importance of ATX in the function of various systems, it has been implicated in many human diseases, some of which will be discussed here.

### 4.1. Autotaxin and malignancies

Before being identified as a phospholipase that catalyzes the production of LPA, ATX was originally reported as a substance present in the supernatant of melanoma cells that induces their migration (28). Serum ATX levels have been reported to be elevated in patients with various malignancies, including hepatocellular carcinoma (29) and follicular lymphoma (25). Serum ATX levels may also be useful in assessing disease progression. For example, in patients with hematological malignancies, ATX levels in cerebrospinal fluid is increased in patients who have malignant cells within the central nervous system (30).

### 4.2. Autotaxin and liver diseases

Serum ATX levels increases in various liver diseases, such as chronic hepatitis C (31) and non-alcoholic fatty liver disease (32). Serum ATX correlates with the histological staging of liver fibrosis and is approved as a marker for liver fibrosis in Japan (33, 34).

### 4.3. Autotaxin and cardiovascular diseases

By producing LPA and mediating platelet activation, ATX also plays an important role in atherosclerosis and cardiovascular diseases. For example, ATX is overexpressed in the cardiac tissue of patients with acute myocardial infarction (AMI) and is involved in the induction of inflammation after AMI (35). LPA is also involved in cardiac remodeling following AMI.

### 4.4. Autotaxin, idiopathic pulmonary fibrosis, and systemic sclerosis

ATX is also implicated in fibrosis, and ATX levels are increased in lungs of patients with idiopathic pulmonary fibrosis (IPF). The ATX-LPA axis may be a potential therapeutic target in IPF, and clinical trials of ziritaxestat, an ATX inhibitor, are being conducted (9, 36). The ATX-LPA axis is also involved in skin fibrosis in systemic sclerosis (37), and a recent clinical trial of ziritaxestat for patients with early diffuse cutaneous systemic sclerosis indicated that inhibition of ATX may improve skin symptoms (38).

### 4.5. Autotaxin and rheumatoid arthritis

The ATX-LPA axis is also involved in pathogenesis of autoimmune diseases, including rheumatoid arthritis (RA) (15). TNF-α, a key cytokine in the pathogenesis of RA, induces expression of ATX in synovial fibroblasts (39), and LPAR1 and ATX are highly expressed in the synovium of patients with RA (39, 40). The ATX-LPA axis has been reported to contribute to the pathogenesis of RA by inducing infiltration of immune cells to joints and may promote differentiation of Th17 cells in the synovium (15, 40).

## 5. Autotaxin and systemic lupus erythematosus

ATX is also expressed by cells of the immune system. In the immune system, ATX and LPA exert various effects––regulating the development, function, and migration of various immune cells––and there is growing evidence that ATX may also play a role in the pathogenesis of SLE. In the following section we will discuss the role of ATX in the immune system and in SLE.

### 5.1. Autotaxin, type I interferons, and plasmacytoid dendritic cells

Production of type I interferons by plasmacytoid dendritic cells (pDCs) plays a central role in the pathogenesis of SLE (41–44). Type I interferons produced by pDCs induce the differentiation of dendritic cells (DCs). It also enhances the ability of DCs to present autoantigens to T cells (44, 45). Type I interferons also promote the maturation of B cells and production of autoantibodies (46–48), and immune complexes further induce the activation of pDCs and production of type I interferons (Figure 1C). Among peripheral blood immune cells, the expression of *ENPP2*, which encodes ATX, is highest in pDCs (49, 50), and several lines of evidence suggest that there is an association between the ATX-LPA axis and production of type I interferons by pDCs in SLE.

First, type I interferons are involved in the production of ATX. The induction of ATX by interferons has been reported in various cell types, including THP-1 cells, human monocyte-derived DCs, and human monocytes. The production of type I interferons upon TLR stimulation plays a critical role in the induction of ATX, and blocking interferons inhibits this process (18).

In addition, the expression of *ENPP2* is increased in pDCs of patients with SLE, especially those with high disease activity (49). However, this is not specific to SLE and is seen in other inflammatory diseases, including COVID-19 infection (50). In single-cell RNA-seq analysis of pDCs, clusters enriched in type I IFN transcripts expressed *ENPP2*, as well as *SLC7A11* and *MYO1E*. *ENPP2* and *SLC7A11* were also expressed in pDCs obtained from kidney biopsies of patients with lupus nephritis. *In vitro* studies suggest that *SLC7A11* induces the expression of *MYO1E* and *ENPP2* during pDC activation and that ATX is necessary for production of IFN-α and TNF. Therefore, ATX may play a critical role in activated pDCs that are directly involved in SLE pathogenesis at the site of inflammation (51). LPA has been reported to modulate the activity of TCF4, a transcription factor essential for pDC development (16).

Other studies have suggested an interaction between the ATX-LPA axis and pDCs in SLE. In our recent weighted gene co-expression network analysis (52) of transcriptome data of pDCs from patients with SLE, *ENPP2* belonged to a module (a group of genes with high correlation in expression patterns) enriched in genes involved with interferon signaling (49). Furthermore, this module included genes whose expression are influenced by single-nucleotide polymorphisms (SNP) associated with SLE in genome-wide association studies. For example, this module included *RASGRP3*, whose expression is increased in pDCs of patients with the SLE risk allele at rs13425999. Although it is not clear whether there is a direct causal relationship between the expression of those genes with ATX, it suggests that ATX may be involved in the connection between genetic risk factors of SLE and disease pathogenesis (49).

Furthermore, among patients with SLE, rs10980684, an intron SNP located in the *LPAR1* gene, is associated with anti-Ro and anti-Sm antibody positivity, which are known to be associated with high serum levels of interferon α. Among patients with anti-Ro and anti-Sm antibody positivity, the T allele at rs10980684 was associated with high serum levels of interferon α (53).

Together, these studies suggest that there is an association of the ATX-LPA axis in pDCs with type I interferons and that genetic factors of SLE play a possible role in this process.

### 5.2. Autotaxin and other myeloid cells

Other DC subsets and macrophages are also involved in the pathogenesis of SLE (44, 54), and the ATX-LPA axis is also critical in the regulation of those cells. For example, expression of ATX in macrophages increases upon activation in both humans and mice. LPA has been reported to increase the production of proinflammatory cytokines, such as TNF. LPA enhances the ability of human macrophages to stimulate T cells (55), and knockdown of ATX expression in mouse macrophages impairs their migratory capacity and ability to activate T cells (56). It has also been reported that LPA modulates the differentiation of monocyte-derived DCs (57). The altered expression of ATX, and thus LPA, in SLE may contribute to the altered function of macrophages and DCs.

### 5.3. Autotaxin and lymphocytes

In lymph nodes, high endothelial venules express ATX at high levels (58). Activated T cells express receptors for ATX, and transendothelial migration of T cells is enhanced by the LPA produced by ATX on high endothelial venules (58, 59). Consistent with this, LPAR2-deficient CD4+ cells exhibit a defect in early intranodal migration (60). In lymph nodes, ATX is also expressed by stromal cells, and the ATX-LPA axis, along with other chemokines, is also involved in the regulation of T cell migration in the paracortex (61). In addition to its role in the migration of lymphocytes, the ATX-LPA axis has been reported to play a role in the formation of immune synapses in CD8+ cells (62).

The ATX-LPA axis is also important for B cells. Some *in vivo* studies suggest that B cells may also be involved in production of ATX during inflammation (63). Furthermore, *via* signaling through LPAR5, LPA has been reported to inhibit B cell receptor signaling (64).

Various abnormalities involving the ATX-LPA axis have been reported in lymphocytes of patients with SLE (65). *LPAR3* has been reported to be upregulated in CD4+ and CD8+ T cells of patients with SLE (66). In addition, in a transcriptome analysis of B cells from patients with SLE and healthy controls, differentially expressed genes were enriched in “chemotaxis and lysophosphatidic acid signaling *via* GPCRs” (67).

### 5.4. Autotaxin–lysophosphatidic acid axis in other organ systems

Cardiovascular diseases are a major cause of mortality among patients with SLE in many cohorts (68, 69). Steroids used for the treatment of SLE promotes progression of atherosclerosis. SLE is often associated with anti-phospholipid syndrome that is characterized by the presence of anti-phospholipid antibodies and thrombosis (70). Consistent with the role of ATX in the activation of platelets, the proportion of ATX+ platelets were reported to be higher in patients with SLE, especially those with a history of thrombosis, compared with that in healthy controls (71). Thus, ATX might be useful as a marker for thrombosis in SLE.

The ATX-LPA axis is also involved in neuropathic pain (72, 73). Among patients with SLE, pain is often a significant burden, even among those whose disease activity is not high (74, 75). It may be possible that ATX is involved in pain in SLE.

## 6. Autotaxin as a biomarker for systemic lupus erythematosus

Reflecting the potential role of the ATX-LPA axis in SLE pathogenesis, various studies have addressed the potential role of ATX as a biological marker in SLE (Table 1).

**Table 1:** Studies assessing autotaxin or *ENPP2* in clinical samples from patients with systemic lupus erythematosus.

	Sample	Results	References
1	Serum ATX concentrations	The concentration of ATX was higher in patients with untreated SLE compared to that of HC in both females and males.	(49)
2	mRNA expression level of *ENPP2* in immune cells	*ENPP2* expression was higher in pDCs of patients with SLE compared to that in HC.	(49)
3	Serum ATX concentrations	ATX levels were higher in patients with lupus nephritis compared to those with other glomerulonephritis, such as diabetic nephropathy.	(27)
4	Urinary ATX/Cre concentrations	Urinary ATX/Cre concentrations were higher in patients with lupus nephritis compared to those with HC.	(76)

ATX, autotaxin; HC, healthy controls; pDCs, plasmacytoid dendritic cells; SLE, systemic lupus erythematosus.

### 6.1. Autotaxin in serum of patients with systemic lupus erythematosus

Consistent with the increase in *ENPP2* mRNA expression levels observed in patients with SLE, the concentration of ATX in the serum is increased in patients with active, untreated SLE compared with that in healthy controls (49). It has also been reported that ATX levels are increased in the serum of patients with lupus nephritis compared with those in patients with other glomerulonephritis, such as diabetic nephropathy and membranous nephropathy (27). The level of ATX in the serum inversely correlates with the dosage of steroids in patients with lupus nephritis (27), and ATX serum levels may decrease upon treatment with steroids (26). Therefore, the effect of treatment must be considered based on ATX serum levels.

### 6.2. Autotaxin in urine of patients with systemic lupus erythematosus

The concentration of ATX in urine shows correlation with various parameters associated with kidney injury, such as the concentration of proteins, N-acetyl-β-d-glucosaminidase, and α1-microglobulin in the urine (76). Urinary ATX/Cre concentrations were higher in patients with lupus nephritis compared to those in controls (76). The concentration of urinary ATX is also increased in patients with membranous nephropathy (76) and active sarcoidosis (77). Therefore, although it may not be disease specific, urinary ATX levels may serve as a potential marker for lupus nephritis.

## 7. Discussion

SLE is a heterogenous disease, and to provide better care for patients with SLE, there is an eminent for biological markers to assess the pattern and extent of organ involvement and to predict treatment response. The ATX-LPA axis is involved in various biological processes, including immune responses. Recent evidence suggests that the ATX-LPA axis is associated with the abnormal production of type I interferons in pDCs that characterizes SLE (49, 51); thus, the ATX-LPA axis may play a critical role in SLE pathogenesis, and ATX may serve as a potential biomarker.

In the immune system, expression of ATX is high in pDCs and induced by type I interferons (18). ATX may be a marker of activated pDCs that are involved in the pathology of SLE, and in addition to acting as a marker for activated pDCs, ATX may directly be involved in the activation of pDCs (51). Reflecting this, expression of *ENPP2* is high in pDCs of patients with SLE, especially those with high disease activity (49), and ATX concentrations increase in the serum of patients with untreated SLE (49) and lupus nephritis (27). The concentration of ATX is also increased in the urine of patients with lupus nephritis (76). Although the ATX concentration increases observed in serum and urine are not specific to SLE, it may be possible that ATX serves as a useful marker for assessing disease activity and the pattern of organ involvement. Furthermore, biologic therapies that directly inhibit type I interferon signaling, such as anifrolumab, have recently become available for the treatment of SLE (78), and novel markers to identify patients who could benefit the most from those therapies need to be identified (79). ATX may serve as a useful marker in this aspect, as it can be measured with a commercial automated immunoassay analyzer (80), which may be more convenient than measuring the expression of interferon-associated genes with quantitative polymerase chain reaction as performed in some clinical studies (78). More studies are needed to assess the potential role of ATX in predicting treatment response.

In conclusion, the ATX-LPA axis plays a critical role in the pathogenesis of SLE and is associated with the production of interferons by pDCs. ATX may serve as a potential marker for assessing disease activity, the pattern of organ involvement, and predicting treatment responses. Thus, further investigation of the role of ATX in these aspects are warranted.

## Author contributions

YT, HS, TS, and KF contributed to the literature review and drafting of the manuscript. All authors contributed to the article and approved the submitted version.

## Conflict of interest

The authors declare that the research was conducted in the absence of any commercial or financial relationships that could be construed as a potential conflict of interest.

## Publisher’s note

All claims expressed in this article are solely those of the authors and do not necessarily represent those of their affiliated organizations, or those of the publisher, the editors and the reviewers. Any product that may be evaluated in this article, or claim that may be made by its manufacturer, is not guaranteed or endorsed by the publisher.
